# Robust Sounds of Activities of Daily Living Classification in Two-Channel Audio-Based Telemonitoring

**DOI:** 10.1155/2013/696813

**Published:** 2013-04-22

**Authors:** David Maunder, Julien Epps, Eliathamby Ambikairajah, Branko Celler

**Affiliations:** School of Electrical Engineering and Telecommunications, The University of New South Wales, Sydney, NSW 2052, Australia

## Abstract

Despite recent advances in the area of home telemonitoring, the challenge of automatically detecting the sound signatures of activities of daily living of an elderly patient using nonintrusive and reliable methods remains. This paper investigates the classification of eight typical sounds of daily life from arbitrarily positioned two-microphone sensors under realistic noisy conditions. In particular, the role of several source separation and sound activity detection methods is considered. Evaluations on a new four-microphone database collected under four realistic noise conditions reveal that effective sound activity detection can produce significant gains in classification accuracy and that further gains can be made using source separation methods based on independent component analysis. Encouragingly, the results show that recognition accuracies in the range 70%–100% can be consistently obtained using different microphone-pair positions, under all but the most severe noise conditions.

## 1. Introduction

### 1.1. Home Telemonitoring

The devotion to one's parents has been displayed in various cultures throughout time and, as such, has usually included the care of one's parents in old age. In colonial times, the care of frail aged persons was primarily the responsibility of the family. Today in the West, elderly people are primarily cared for in hospitals, nursing homes, or by their families.

The United Nations predicts that by 2100, 28.1% of the world population will be aged 65 years or older, compared with 10.0% in 2000 and 6.9% in 1990 [1]. The resulting increased demand on the health system, coupled with decreasing taxpayer support and fewer younger people to care for the elderly, will introduce significant pressure on aged care services. The main needs of these services are monitoring and supporting elderly people. For example, the fifth highest cause of death for elderly people is falls [2], and while the falls themselves may not be easily preventable, in many circumstances the deaths following them are, with appropriate monitoring and support. Consequently, significant research interest has been focused towards home-telecare solutions allowing elderly people to live safely and independently in their homes. 

In recent years, it has been suggested that sound “signatures” are well suited to automated telemonitoring of elderly people and superior to video cameras from the perspective of privacy [3]. Telemonitoring using sound signatures is a relatively less explored area in the literature, in comparison with other techniques such as gait parameters and posture and motion information.

The main methods for monitoring elderly people are the “Health Smart Home,” wearable devices, or a combination of the two. The first Health Smart Home [4] used magnetic switches between doors, infrared sensors, and sound sensors to monitor which room or part of a room the person was in and the activity they were undertaking. In [5], infrared sensors and magnetic switches were used to monitor the activities of the subject and compare them with expected behaviour. The main shortcomings of the Health Smart Home are the restriction to monitoring only nonbiomechanical parameters, its inability to monitor elderly subjects outside their home, and the need to distinguish the elderly person from others in the home.

Wearable devices are designed to measure biomechanical/physiological parameters and can be attached to clothing, worn either as jewellery or as a separate device [6]. Pedometers, foot-switches, and heart rate measurements can monitor the level of a person's daily activity and energy expenditure. Accelerometers measure the acceleration along the *x*, *y*, and *z* axes and can measure dynamic and static activities over a short or long period of time. Gyroscopes measure the Coriolis acceleration from rotational angular velocity and can therefore measure changes in the wearer's posture. The major disadvantage of wearable devices is that they are not able to be used by people who are incapable, physically or mentally, of operating and maintaining them.

Investigations into telemonitoring using sound sensors have been performed by a French consortium of the CLIPS and TIMC laboratories, who developed a five-microphone system [3, 7, 8]. The main shortcoming of this work is the specialised wiring in the dedicated test apartment [9]. In addition, sound files in their database were recorded in a controlled environment and the noise sources mixed in artificially. Nevertheless, this group has demonstrated the feasibility of classifying a range of different household sounds [6, 8–10].

Source separation is a problem in which a sound sensor receives a number of component sources, and the goal is to determine the original component sources. One technique for single-microphone source separation is to express each separated source as a weighted sum of subbands (refiltering) [11]. A newer algorithm, which uses sub-band independent component analysis, has been proposed where each of the frequency bins is composed of a linear combination of the corresponding frequency bins. This technique is combined with a beamformer which estimates the direction of arrival [12]. In [13], an acoustic event classification system is described which applies temporal ICA on a single-microphone audio signal to determine the temporal window during which features should be extracted, resulting in improved system performance. In this context, ICA was used as a method segmenting different sound sources rather than for separating them in an acoustic sense using multiple microphones.

A variant of the bionic wavelet transform, an adaptive wavelet transform derived from a nonlinear auditory model of the cochlear, has been applied to the task of single-microphone speech enhancement [14]. A novel prefilter has also been developed and uses temporal and simultaneous masking thresholds to shape the speech spectrum in order to obtain a better estimate of the autoregressive coefficients that characterize the speech spectrum [15].

### 1.2. Robust Sounds of Daily Life Classification from Two-Channel Audio

In many instances, the objective of telemonitoring is to form a model of the “activities of daily life” [6], so that deviations from this model can be used to automatically prompt for help. In sound-based telemonitoring, the aim is similar: to distinguish between different “sounds of daily life” (SDLs), which may correspond to some or all of the activities of daily life. 

**Figure 1:**
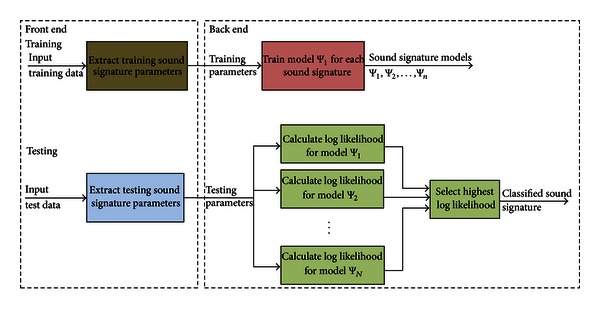
Typical configuration for a “sounds of daily life” classification system, which we refer to herein as the baseline system. Classified sound signatures can then be modelled for telemonitoring purposes.

Figure 1 depicts a generic sound signature classification system. Extraction of sound signature parameters occurs at the “front-end,” where information putatively useful for differentiating between sound signatures is obtained from the microphone signal. As a precursor to the front-end, clearly it would be desirable to detect the presence or absence of a relevant SDL. Similar approaches have been adopted in [3, 7, 8, 16] where the energy of the most discriminating bands from the discrete wavelet transform (DWT) were used to distinguish between an event and noise. For example, to distinguish between a kettle sound and a door slap, energy may be a useful parameter, as the energy in the kettle sound would be significantly lower than that of the door slap (assuming the microphones were equidistant from each sound source). Sound modelling and classification are then conducted in the back-end. Since both microphones will normally contain both signal and noise components, a two-stage system is proposed, with the first stage attempting to determine estimates for the signal and noise. 

### 1.3. Source Separation

In general, multimicrophone sensing of sounds of daily life in realistic environments is likely to produce individual microphone signals comprising the superposition of the sound of daily life and other background noises. A related problem is known as the “cocktail party effect,” in which a number of people are speaking and a listener desires to listen exclusively to one of them. In a telemonitoring application, the objective is somewhat different: to separate the sound source from the background noise source, in order to classify it more accurately. Suppose that we define each microphone signal *x*_*i*_[*n*], *i* = 1,2,…, *M*, as a linear combination of the unknown sources *s*_*j*_[*n*], *j* = 1,2,…, *N*,

(1) [x1[n]x2[n]⋮xm[n]]=[a11a12⋯a1pa21a22⋯a2p⋮⋮⋱⋮am1am2⋯a  mp  ][s1[n]s2[n]⋮sp[n]].

Independent component analysis (ICA) assumes that the input source distributions are non-Gaussian and independent. ICA estimates a matrix **B**, which is the inverse of **A** = (*a*_*ij*_)_*M*×*N*_ (in the case *M* = *N*) from (1), to separate the sources [17].

ICA uses the central limit theorem result, which states that a linear combination of non-Gaussian independent sources will converge to a Gaussian distribution. Therefore, the distributions of the sensor signals *x*_*i*_[*n*] will be more Gaussian than those of the sources *s*_*i*_[*n*]. ICA estimates the matrix **B**, which when applied to the sensors *x*_*i*_[*n*] attempts to minimise the Gaussianness of the estimated separated sources. This leads to ${\hat{s}}_{i}[n],\;\;i=1,2,\ldots ,N$ estimated separated sources. Various metrics are available for measuring a source's Gaussianness, including kurtosis and negentropy [17].

In the context of this work, the sensor signals are recorded by *N* = 2 microphones *m*_1_[*n*] and *m*_2_[*n*]. Each microphone is composed of a linear combination of the sound of daily life source *s*_SDL_[*n*] and noise source *s*_noise_[*n*]:

(2) $$\begin{array}{c} \left[\begin{array}{c} m_{1}\left[n\right]\\ m_{2}\left[n\right] \end{array}\right]=\left[\begin{array}{cc} a_{11} & a_{12}\\ a_{21} & a_{22} \end{array}\right]\left[\begin{array}{c} s_{\text{SDL}}\left[n\right]\\ s_{\text{noise}}\left[n\right] \end{array}\right]. \end{array}$$

ICA estimates the matrix **B**, which minimizes the Gaussianness of *s*_sep1_[*n*] and *s*_sep2_[*n*] and determines estimates for the separated sources:

(3) $$\begin{array}{c} \left[\begin{array}{c} s_{\text{sep}1}\left[n\right]\\ s_{\text{sep}2}\left[n\right] \end{array}\right]=\left[\begin{array}{cc} b_{11} & b_{12}\\ b_{21} & b_{22} \end{array}\right]\left[\begin{array}{c} m_{1}\left[n\right]\\ m_{2}\left[n\right] \end{array}\right]. \end{array}$$

There are two problems encountered using ICA:

1. ICA only determines the separated sources up to a scaling factor. The matrix **B** could be multiplied by a scaling factor without changing the Gaussianness of *s*_sep1_[*n*] and *s*_sep2_[*n*];
2. ICA separates the sources, but does not identify which is the SDL and which is the noise source.

The focus of this paper is the classification of sounds of daily life (SDLs) from multiple microphones of unknown location, in the presence of noise. If a range of SDLs can be detected, a distress situation such as a thud, which could correspond to a fall or a cry for help, could be discovered and appropriate medical care sent to the house. In addition, a model of elderly people's daily activities can be developed from which important information about their functional health status derived. This work is more portable than previously reported systems that employ fixed microphones [3, 6–10], employing two microphones which can be easily installed in a private residence, and data has been recorded in realistic scenarios in the presence of typical background noises. 

**Figure 2:**
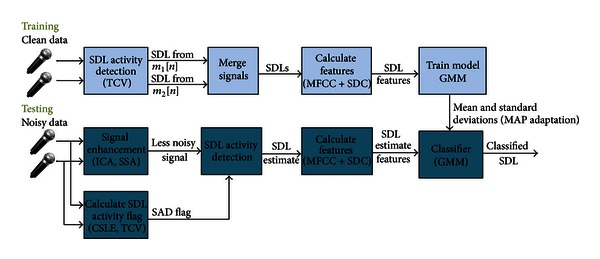
Proposed configuration for a robust “sounds of daily life” classification system, based on multiple microphones.

In particular, we develop methods for addressing the problems of identifying the *s*_SDL_[*n*] signal following source separation and sound activity detection, as shown in Figure 2, and evaluate these on a large and realistic new multi-microphone database. The work builds on and substantially extends earlier work on sound activity detection for robust sounds of daily life classification [18].

## 2. Proposed Source Selection Methods

Due to the prevalence of independent component analysis among source separation problems, we also adopt this method. To overcome the problem of ICA determining the sources only up to a scaling factor, the separated sounds of daily life are herein normalized to have a maximum value of 1. This makes the data consistent. There are a number of ways to overcome the problem of identifying the *s*_SDL_[*n*] signal following source separation, each with its advantages. The main approach adopted herein is to determine which separated source has the least noise; however, it may not always be straightforward to identify which signal components are sounds of daily life and which are noise. Five possible solutions to this problem are proposed as follows. 

### 2.1. Source Selection with *a Priori* SNR

If prior knowledge is available about which microphone has the best SNR, the separation matrix can be analysed to determine which separated source has the superior SNR. Without loss of generality, assume that *m*_1_[*n*] has the best SNR. If it does not, it can be swapped with *m*_2_[*n*] to ensure it does. If  |*b*_11_|^2^ > |*b*_12_|^2^, the first separated source contains more data from the microphone with the best SNR and therefore has the least noise in it. Alternatively, if | *b*_11_|^2^ < |*b*_12_|^2^, the second separated source has the smaller amount of noise:

(4) $$\begin{array}{c} {\hat{s}}_{\text{SDL}\_\text{Apriori}}\left[n\right]=\{\begin{array}{cc} s_{\text{sep}1}\left[n\right] & \text{if}\;\;{\left|b_{11}\right|}^{2}>{\left|b_{12}\right|}^{2}\\ s_{\text{sep}2}\left[n\right] & \text{if}\;{\left|b_{11}\right|}^{2}<{\left|b_{12}\right|}^{2}. \end{array} \end{array}$$

The main shortcoming of this algorithm is that it requires prior information as to which microphone has the best SNR. This requirement is addressed by the three methods given in the ensuing subsections.

### 2.2. SNR Estimation Source Selection Using Microphone with Best SNR

This method extends the first method and automates the process of determining which microphone has the least amount of noise. A related problem is the determination of whether a sound is present or not, which we term sound activity detection (SAD). For each of the microphone signals, the SAD algorithm is applied (see Section 3), the SDL and noise segments are found using a reliable SAD algorithm—we used CSLE—and an estimate of the SNR determined using the following formula:

(5) $$\begin{array}{c} \text{S}\hat{\text{N}}{\text{R}}_{\text{BestMic}}=\frac{\sigma_{\text{SDL}+w}^{2}}{\sigma_{w}^{2}}, \end{array}$$

where *σ*_SDL+*w*_^2^  is the average SDL (signal plus noise) power and *σ*_*w*_^2^ is the average noise power, estimated from segments of the signals suspected to contain SDLs and noise, respectively.

The microphone with the largest SNR is found and (5) is applied. This method has the advantage of not requiring prior information. In addition, due to the varying microphones, noise and SDL locations, the microphone with the least noise may change. However, the main shortcoming is that if the SAD is not sufficiently accurate, the microphone with the least amount of noise could be incorrectly selected which will result in not choosing the SDL correctly.

A variant of this method is

(6) $$\begin{array}{c} \text{S}\hat{\text{N}}{\text{R}}_{\text{LeastNoise}}=\frac{1}{\sigma_{w}^{2}}, \end{array}$$

where *σ*_*n*_^2^ is the average noise power. This means that the SAD algorithm only needs to classify noise frames correctly (its performance is unaffected by noise frames which are classified as SDL frames).

### 2.3. Source Selection Using Separated SDL with Best SNR

The SNR estimates from the previous subsection extract information from the input microphone signals to determine which separated source has the best SNR. The methods detailed in this section use information from the separated sources to determine which has the least amount of noise. This approach obtains an SNR estimate by applying (8) to each separated source and determines which has the best SNR:

(7) s^SDL_BestSNR[n] ={ssep1[n]  if  SNR(ssep1[n])>SNR(ssep2[n])ssep2[n]  if  SNR(ssep1[n])<SNR(ssep2[n]).

### 2.4. Source Selection Using Separated SDL with Least Noise

This method is similar to that of Section 2.3, however only the noise segments are used. The SNR estimate is determined using $\text{S}\hat{\text{N}}{\text{R}}_{\text{LeastNoise}}$, which is then used as the SNR estimate in (7), to produce a separated source ${\hat{s}}_{\text{SDL}\_\text{LeastNoise}}[n]$. As with the calculation of $\text{S}\hat{\text{N}}{\text{R}}_{\text{LeastNoise}}$, this technique means the SAD classification does not need to be as stringent.

### 2.5. SNR Steepest Ascent (SSA) for Improvement of the Unmixing Matrix

As discussed in Section 2.1, in some situations it is possible to use sound activity detection to identify regions of the signal for which there is noise only or sound and noise together. For the special case of two-source separation, this information can be used to estimate improved estimates of the unmixing matrix entries. Consider the separated SDL from (7) and without loss of generality, assume that *s*_sep1_[*n*] is the separated SDL following source selection. We define the SNR of the separated SDL as follows:

(8) SN^RSDL_SA(b11,b12) =(1/|{nK,nK+1,…,nG}|)∑n∈{nK,nK+1,...,nG}(b11m1[n]+b12m2[n])2(1/|{nL,nL+1,…,nH}|)∑n∈{nL,nL+1,...,nH}(b11m1[n]+b12m2[n])2,

where {*n*_*K*_, *n*_*K*+1_,…, *n*_*G*_} are the SDL and noisy sample indices, and {*n*_*L*_, *n*_*L*+1_,…, *n*_*H*_} are the noisy sample indices.

As *b*_11_ and *b*_12_ are varied, the SNR varies. The SNR space may exhibit an arbitrary number of peaks if both parameters are varied. If one of the parameters is constant and the other is varied, only one peak should exist.

**Figure 3:**
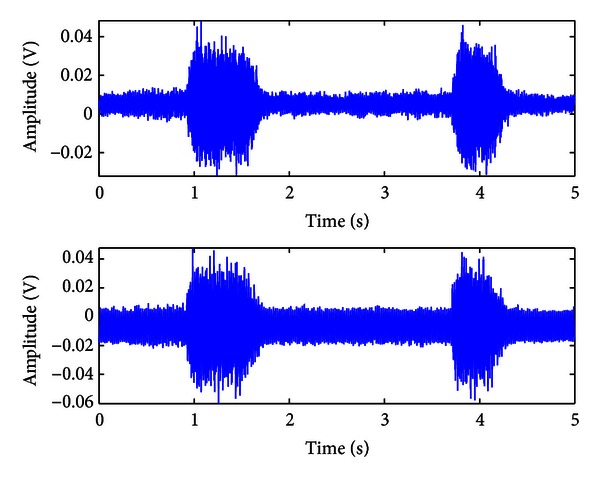
Example microphone signals for a phone ringing (sound of daily life—higher energy segments) with the radio on (background noise—lower energy segments).

**Figure 4:**
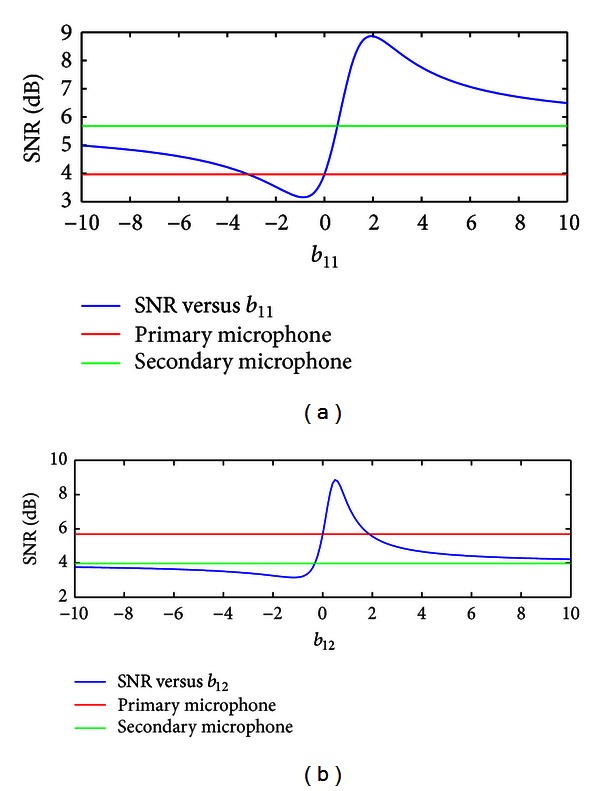
Plot of SNR as the unmixing matrix coefficient *b*_12_ is varied, for the two channels shown in Figure 3. The first (a) fixes the primary microphone as constant and adds the secondary microphone scaled by *b*_12_. The second (b) fixes the secondary microphone as constant and adds the primary microphone scaled by *b*_12_. Both yield approximately the same peak.

Figure 4 depicts the variation in SNR for the two microphone signals in Figure 3 as a function of  *b*_12_. The first plot shows *m*_1_[*n*] set as the primary and *m*_2_[*n*] set as the secondary microphone. The second swaps *m*_1_[*n*] and *m*_2_[*n*] (which is equivalent to not swapping the microphones, but holding *b*_12_ constant and varying *b*_11_). Both plots yield the same peak SNR, suggesting that the algorithm only needs to be performed for one combination of the microphone signals. 

Attempting to find the SNR over an entire range of suitable *b*_12_ values is computationally burdensome. Therefore, a steepest ascent approach is proposed. Let *m*_best_[*n*] and *m*_worst_[*n*] be the microphone signals with the best and worst SNRs, respectively. If *b*_11_ = 1 and *b*_12_ = 0 then

(9) $$\begin{array}{c} s_{\text{SDL}}\left[n\right]=b_{11}m_{\text{best}}\left[n\right]+b_{12}m_{\text{worst}}\left[n\right]. \end{array}$$

The algorithm can be initialized by *b*_11_ = 1  and *b*_12_ = 0, that is, the initial separated SDL will have the same SNR as that of the microphone with the best SNR. The approach adopted is to vary the separated source by adding or subtracting a scaled version of the microphone with the worst SNR for the purpose of improving the SNR of the separated source,

(10) $$\begin{array}{c} b_{12}=b_{12}+\mu \frac{\partial \text{SN}{\text{R}}_{\text{SDL}}}{\partial b_{12}}, \end{array}$$

where

(11) ∂SNRSDL∂b12=∂∂b12(1/N)∑n∈S(b11mbest[n]+b12mworst[n])2(1/M)∑n∈N(b11mbest[n]+b12mworst[n])2  =Q−R((1/M)∑n∈N(b11mbest[n]+b12mworst[n])2)2,Q=1M∑n∈N(b11mbest[n]+b12mworst[n])2   ×21N∑n∈S(b11mbest[n]+b12mworst[n])mworst[n],R=1N∑n∈S(b11mbest[n]+b12mworst[n])2  ×21M∑n∈N(b11mbest[n]+b12mworst[n])mworst[n].

Since the vector (1, ∂SNR_SDL_/∂*b*_12_) “points” in the direction of steepest ascent in the (*b*_11_, *b*_12_) space, adapting the parameter *b*_12_ in this direction will increase the SNR of the separated source.

In (10), the adaptation parameter *μ* is constant and determines the convergence of the algorithm. If *μ* < 0, *b*_12_ approaches a value that yields the smallest SNR. If  *μ* > 0, *b*_12_ approaches a value yielding the largest SNR. Additionally, if *μ* is small, the rate of convergence will be slow. If *μ* is large, there is possible instability in the result—although both issues are mitigated through adapting *μ*, as outlined below.

**Figure 5:**
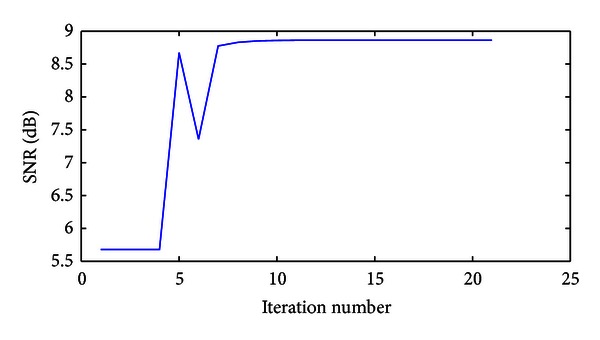
Example SNR versus iteration number for the SNR steepest ascent method. Overshoot is detected at iteration 6, at which point the value of *μ* is decreased to minimise the likelihood of the algorithm overshooting again.

The SNRs in the database of SDLs are very diverse. As a result, for some values of *μ*, the algorithm will converge quickly, some will converge slowly, and others will overshoot and miss the peak completely. To overcome this, an adaptive *μ* is implemented. The value of *μ* begins with a high value and when overshoot is detected (which is found by a negative change in SNR), the algorithm backtracks by one iteration and *μ* is decreased. A suitable method for achieving this, determined empirically, is multiplying *μ* by a factor of 0.1 each time an overshoot is detected.  Figure 5 shows how the SNR evolves in each iteration of the algorithm for the example recorded in Figure 3.

**Figure 6:**
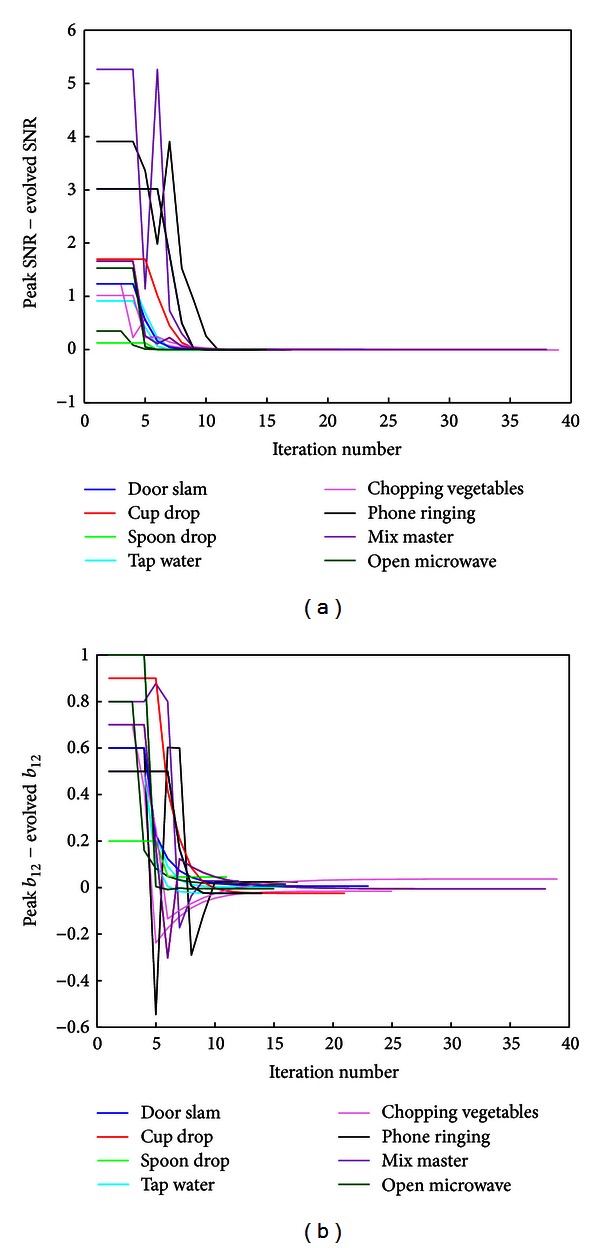
Convergence behaviour of the SSA algorithm: differences between the optimum SNR and evolved SNR (a) and optimum *b*_12_ value minus evolved *b*_12_ (b), for various example sounds of daily life (as indicated) with background radio noise at pleasant listening level, using microphones 1 and 3. Two SDLs from each sound class were chosen at random.

**Figure 7:**
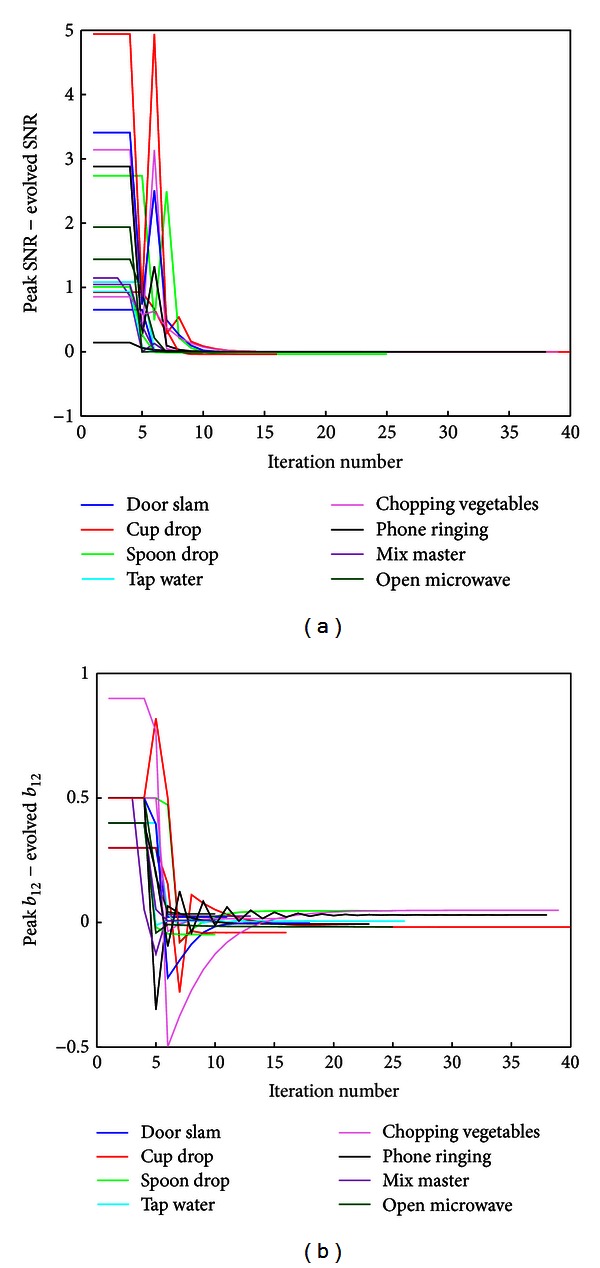
Difference between best SNR and evolved SNR (a) and best *b*_12_ value minus evolved *b*_12_ (b), for the database with a male speaker in the background, using microphones 1 and 3.

It was investigated whether the algorithm would converge in less time than if the optimum SNR was found through an exhaustive search. Recordings from the databases for different SDLs with radio noise were used. Two recordings were chosen at random, for each of the eight SDLs from the radio at pleasant listening level and male speaker databases, using microphones 1 and 3.  Figure 6 shows a plot of the difference between the best SNR (which was determined by an exhaustive search across *b*_12_ ∈ [−10,10] and the SNR) which evolves in the algorithm.  Figure 7 shows the difference between the value of *b*_12_, corresponding to the best SNR value, and the evolved value of *b*_12_. In both cases, all SDLs converge in under 30 iterations. To obtain the same SNR maximum using an exhaustive search over the region *b*_12_ ∈ [−10,10) (assuming we knew which region to select) with a resolution of 0.01, the number of iterations needed would be 2000.

### 2.6. Noise Inverse Steepest Ascent (NISA) for Improvement of the Unmixing Matrix

This method is identical to the SSA method described in Section 2.4, except that here the goal is to minimize the noise power in the pure noise segments. This means that the SAD algorithm only needs to accurately classify the noise frames.

## 3. Sound Activity Detection

The purpose of sound activity detection (SAD) is to identify the location of the SDL in the audio signal (either a raw microphone signal or separated signal) and attempts to remove silence or pure noise components that produce unwanted variability during classification. SAD requires the extraction of features that can distinguish between signal and noise, followed by a classification rule. In these respects, SAD is analogous to voice activity detection in speech processing, and indeed some methods discussed below can be traced to speech processing research.

The spatial separation of the multiple microphones suggests that differences in the relative energies of the sound and background noise in each microphone signal can be exploited to detect the presence or absence of the sound. In array processing, where sensors are placed in a known spatial pattern, eigenvalue methods have been used with success to estimate the bearing of a signal of interest [19]. In the current problem, the spatial pattern of microphone sensors is unknown; however, changes in the composition of the microphone signals due to the presence or absence of a particular source (i.e., the sound of daily life) can still be detected using similar techniques. Motivated by similar considerations, we define the cross-spectral matrix as [18]:

(12) $$\begin{array}{c} C\left[k\right]=\left[\begin{array}{cc} {\left|M_{1}[k]\right|}^{2} & \left|M_{1}\left[k\right]\right|\left|M_{2}\left[k\right]\right|\\ \left|M_{2}\left[k\right]\right|\left|M_{1}\left[k\right]\right| & {\left|M_{2}[k]\right|}^{2} \end{array}\right], \end{array}$$

where *M*_1_[*k*] and *M*_2_[*k*] are the discrete Fourier transforms (DFTs) of the respective microphone signals *m*_1_[*n*] and *m*_2_[*n*]. Under the assumption that the noise is uncorrelated with the signal of interest, it has been shown that the largest eigenvalue of **C**[*k*] comprises signal variance and noise variance terms, while the remaining eigenvalues comprise only noise variance terms [19].

Since **C**[*k*] is a rank one matrix, we consider instead a cross-spectral matrix

(13) $$\begin{array}{c} \overset{-}{C}\left[k\right]=\sum_{m=1}^{M}C_{m}\left[k\right] \end{array}$$

that has been averaged over *M* consecutive frames, where **C**_*m*_[*k*] is the cross-spectral matrix at the *m*th frame calculated using (13), as suggested in [20]. The eigenvalues of this matrix are now [18]

(14) λ1,2=12(|M1[k]|2¯+|M2[k]|2¯)   ±124|M1[k]M2[k]|2¯+(|M1[k]|2¯−|M2[k]|2¯)2.

In the problem at hand, assumptions of uncorrelated signal and noise cannot be made (typical sounds of daily life and noises are discussed in Section 4); however, the choice of frequency or frequency range over which **C**[*k*] is computed can be chosen empirically to minimize the correlation, by selecting frequencies in which the signal and noise are likely to have maximally different spectral characteristics. 

The eigenvalues of the rank one matrix **C**[*k*] are

(15) $$\begin{array}{c} \lambda_{1,2}=\left\{0,{\left|M_{1}[k]\right|}^{2}+{\left|M_{2}\left[k\right]\right|}^{2}\right\}, \end{array}$$

which can be trivially determined from the DFTs of the microphone signals. 

When only the noise source is active, *s*_kitchen_[*k*] = 0. Therefore [18],

(16) $$\begin{array}{c} \lambda_{2}=\{\begin{array}{cc} \left({\left|a_{21}\right|}^{2}+{\left|a_{22}\right|}^{2}\right){\left|s_{\text{noise}}\left[k\right]\right|}^{2} & \\ \;\text{if}\;\text{only}\;\text{noise}\;\text{source}\;\text{is}\;\text{active} & \\ \left(|a_{21}{|}^{2}+|a_{22}{|}^{2}\right){\left|s_{\text{noise}}\left[k\right]\right|}^{2} & \\ \;+\left({\left|a_{11}\right|}^{2}+{\left|a_{12}\right|}^{2}\right){\left|s_{\text{kitchen}}[k]\right|}^{2} & \\ \;+2\left(\left|a_{11}a_{12}\right|+\left|a_{21}a_{22}\right|\right)\left|s_{\text{kitchen}}\left[k\right]\right|\left|s_{\text{noise}}\left[k\right]\right| & \\ \;\;\text{if}\;\text{SDL}\;\text{is}\;\text{occurring}. & \end{array} \end{array}$$

Therefore, one would expect a significant change in *λ*_2_ when the SDL is occurring. Since *λ*_2_ ≥ 0, *λ*_2_ is the largest eigenvalue and the smallest eigenvalue is *λ*_1_ = 0 (in practice *λ*_1_ ≠ 0 but *λ*_1_ ≪ *λ*_2_).

Therefore, a significant change in the largest eigenvalue is expected when an SDL is occurring, in comparison to during the pure noise segments. As the cross-spectral matrix has rank one, it is averaged across several Hamming windowed frames, making it have rank two, allowing the eigen decomposition to be performed. The largest eigenvalues are calculated for each windowed frame average for *k* = 0,1,…, (*N*/2) − 1 where *N* is the length of the DFT.

The quantities $(1/2)\left(\bar{{\left|M_{1}[k]\right|}^{2}}+\bar{{\left|M_{2}[k]\right|}^{2}}\right)$, $4\bar{{\left|M_{1}[k]M_{2}[k]\right|}^{2}}$, and ${\left(\bar{{\left|M_{1}[k]\right|}^{2}}-\bar{{\left|M_{2}[k]\right|}^{2}}\right)}^{2}$, representing terms of (14), were also investigated; however, none of them in isolation matched the performance of *λ*_2_  as a method for sound activity detection.

**Figure 8:**
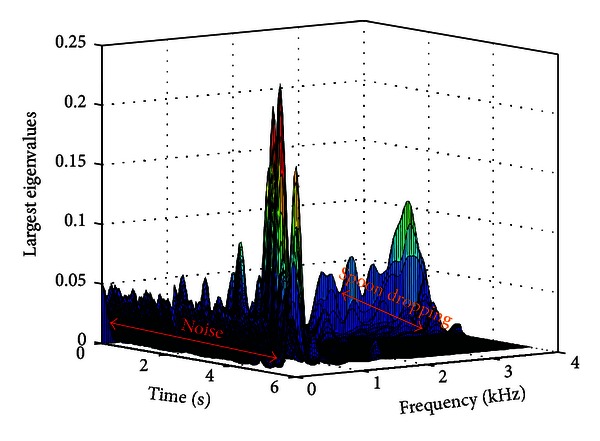
Time-frequency plot of the largest eigenvalues of the averaged cross spectral matrices for a dropping spoon sound in radio noise.

**Figure 9:**
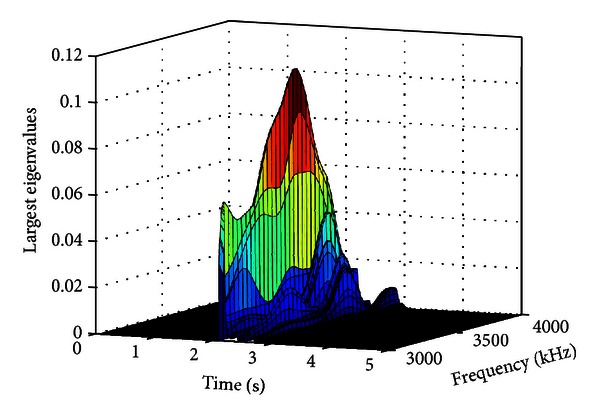
Close-up view of the 3-4 kHz region from the time-frequency plot in Figure 8. This shows a significant non-zero component when the SDL is occurring.

Figure 8 shows a time-frequency plot of the largest eigenvalues for a recording when a spoon was dropped. It was determined empirically that in the 3-4 kHz band, the eigenvalues are approximately zero when only the noise source is active and nonzero when the SDL is occurring for all SDLs in the databases. An example of the 3-4 kHz band is shown for the spoon dropping recording in the presence of radio noise in Figure 9.

An average of the eigenvalues is calculated across the 3-4 kHz band, then converted to a dB scale and normalized to the range [−1,0] dB. This yields an indicator which is small during the pure noise segments and much higher when the SDL is occurring. A threshold was then applied to this largest eigenvalue-based indicator, a method we refer to as baseline SAD. 

**Figure 10:**
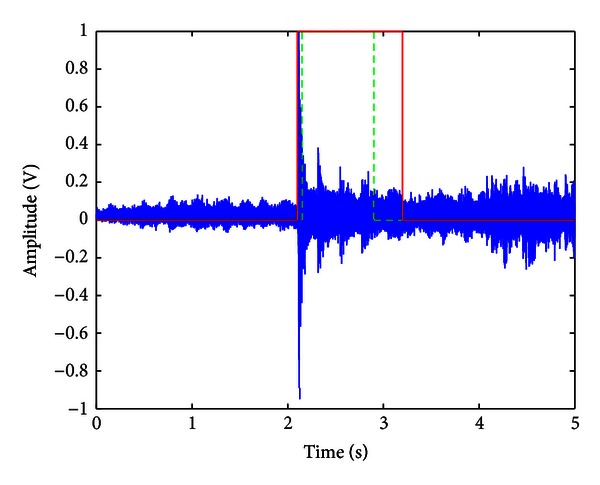
Time-domain waveform comparing an example segmentation (red) with the actual endpoints (green).

An example result of the CSLE algorithm, showing where the SDL was found for a dropping spoon recording, is given in Figure 10. The estimated endpoints are within ±6% of the true endpoints. In the sound files checked across the entire database described in Section 4, similar deviations from the true endpoints were found. 

## 4. Evaluation

### 4.1. Noisy Sounds of Daily Life Database

In previous work by the authors [18], a database of seven sounds of daily life was constructed, in which a single radio was located close to one microphone in the recording environment. While many noise reduction applications have a sound sensor almost completely dedicated to recording the noise, this is not realistic in a general practical environment, as the sound sensors and noise source might be located anywhere. Further, very high accuracies were produced using the database in [18]. Consequently, we decided to investigate more difficult microphone configurations and noises.

**Figure 11:**
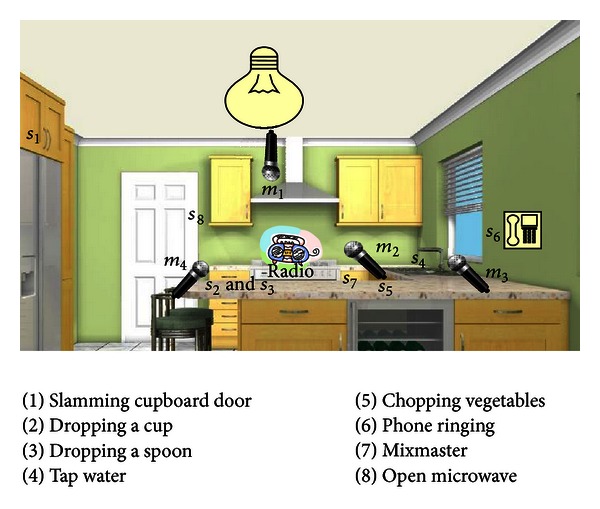
Kitchen configured for data collection to investigate more difficult microphone configurations.

The microphone positions in the database described herein were carefully chosen with neither microphone as close to the radio as in [18]. In addition, the microphones were chosen, where possible, to be near either a power outlet, or the mains electricity (e.g, a light), to realistically evaluate the system's performance, as the microphones would be attached to the mains power if this were to become a commercial product. Microphone 1 was attached to a light, microphones 2 and 3 were mounted near power outlets and microphone 4 was placed diagonally and horizontally opposite microphones 2 and 3, respectively, as there were no more suitable positions near a possible entry to the mains electricity. The configuration for the noisy SDL recordings is depicted in Figure 11.

The eight SDLs recorded comprised a cupboard door slamming (*s*_1_), a cup dropping (*s*_2_), a spoon dropping (*s*_3_), running tap water (*s*_4_), chopping vegetables (*s*_5_), a phone ringing (*s*_6_), a mixmaster operating (*s*_7_), and a microwave door opening (*s*_8_). 

In each instance four channels were recorded to compare system performance in the same conditions for different microphone placements, but a maximum of two microphones were used in any evaluations reported herein.

Each recording had a duration of 5 seconds, and the total duration of the database across all conditions was 2.78 hours (90 training samples × 8 SDLs × 5 seconds = 1 hour. 40 training samples × 8 SDLs × 5 seconds × 4 noise degradations = 1.78 hours). 

System performance in the presence of different noise types is of particular interest. In addition to the radio at pleasant listening level (as in [18]), other noise types recorded were the radio at a very loud volume, a male speaker talking in the background, and data recorded in the presence of rain (note that rain cannot be considered a point source, so does not satisfy the assumptions of CSLE). The estimated SNR ranges, across all conditions, are shown in Table 1.

### 4.2. System Description

The overall system is shown in Figure 2. The noise-corrupted testing data was preprocessed before feature extraction, using either signal enhancement, sound activity detection, or both. Features were then extracted and maximum a posteriori (MAP) adaptation was applied before using the adapted Gaussian mixture model (GMM) to determine the most likely SDL.

Feature extraction was performed using Mel frequency cepstral coefficients (MFCCs), following earlier work showing that importance of magnitude spectrum information in distinguishing between the SDLs [21]. Shifted delta cepstral (SDC) features were also employed, to allow some modelling of the temporal variation of the magnitude spectrum, and based on previous results [21, 22]. 

A GMM classifier with six mixture components was employed, following successes in related applications [21, 23, 24]. This was trained using 10 iterations of the EM algorithm. MAP adaptation of the GMM mean vectors was conducted, to enhance the discrimination between the different sounds of daily life classes over the simpler alternative configuration comprising GMMs trained separately on data from each class. Since, during GMM training, random initialization of GMM parameters was employed, the results reported are the averages across 10 different initializations.

Classifier training was performed using clean sounds of daily life data. If it is trained using a noisy set, it will be unrealistically optimized for a specific spectral and spatial configuration. In any case, mismatch between the training data and real test data is to be expected. The system was trained using data from two microphones. If only one microphone is used, the classifier is biased towards data from that microphone (as it has a unique frequency response and additive noise characteristics). A noise-reduced signal will contain data from two microphones, as suggested by (2). Consequently, even though the SNR of the signal is improved by training using the microphone with the best SNR, the classification performance may not be improved because the unmixed signal passed to the classifier will contain data from both microphones. 

### 4.3. Experiments

All experiments were performed in MATLAB and focused on the eight-class classification accuracy of various components of the proposed system.

1. A comparison of the seven ICA-based methods for source separation among the microphone sources was made in which noise was artificially mixed into clean sounds of daily life data. The objective of this experiment was to eliminate delay between microphones, due to sound propagation, as a source of uncertainty in the results.
2. Combinations of the source separation and sound activity detection methods were tested for one of the microphone configurations of the noisy SDL database, for four different noise configurations. In these experiments, CSLE was selected as the sound activity detection method of choice in most configurations, based on previous work [18].
3. Three of the most promising source separation methods were combined with CSLE and tested for all microphone configurations of the noisy SDL database across all four different noise configurations.

In all tests, the GMM was trained with 30 recordings per sound class (with samples chosen uniformly across the database), and the system was tested using 40 noisy recordings per sound class across the different types of noise. The duration of each recording was 5 seconds. 

## 5. Results and Discussion

### 5.1. Source Separation under Controlled Conditions

As seen in Table 2, under conditions where the noise was artificially mixed into the sounds of daily life, there was a clear advantage to using the steepest descent methods, with the noise inverse steepest ascent method of improving the unmixing coefficients producing the highest accuracies for both conditions tested. This suggests that the SSA and NISA methods may have often been able to find the optimum unmixing coefficients as occurred during the example of Figure 4.

### 5.2. Source Separation and SAD under Noisy Conditions

The advantage of steepest ascent methods over other source separation methods did not seem to be preserved for experiments on the noisy SDL database, in which noises were acoustically mixed in a realistic manner. As seen in Table 3, although the SNR steepest ascent method with CSLE performed best on the Mics 1 and 2 loud radio noise condition, this was by a narrow margin, and various other configurations proved slightly better for other noise conditions. In general, the results of Table 3 primarily demonstrate the importance and effectiveness of sound activity detection for the problem of multichannel SDL classification in noise.

### 5.3. Source Separation and SAD for Different Microphone Configurations under Noisy Conditions

Finally, in Table 4, the accuracies from a selection of the most promising configurations from all conditions are reported, with a view to recommending a good system configuration across all conditions tested. From Table 4, it is clear that sound activity detection using CSLE is the major single contributor to classification accuracy. However, further gains might be achieved using CSLE and ICA based on *a priori* SNR (Section 2.1), whose accuracy either improved on or was roughly comparable with that of CSLE alone.

## 6. Conclusion

This paper has reported the application of source separation and sound activity detection methods to the problem of classifying sounds of daily life for home telemonitoring under realistic noisy conditions using multiple arbitrarily positioned microphones. Overwhelmingly, the experimental evidence shows that the inclusion of an effective sound activity detection method, such as the cross-spectral largest eigenvalue (CSLE) method, in an “activities of daily life” classification system produces large and consistent gains in accuracy, up to 90% in terms of relative reduction in error rate under a range of noisy conditions. Source separation methods, including the ICA variants considered herein, provided at best modest gains. Among these, the ICA_SDL_Apriori_  method, which identifies the two unmixed signals using estimates of their SNRs, seems to perform well, providing relative reductions in error rates of up to 10% in the best case. The overall classification performance on an eight-class task provided accuracies between 70%–100% across three different microphone-pair conditions and three noise conditions, while the most challenging low-SNR loud radio noise condition resulted in accuracies in 40%–50% range, still well above chance level. Future work will examine the long-term performance of this system in the homes of elderly subjects, detecting a range of sounds that are found to commonly occur, and will begin correlating their occurrence with clinical indicators.

## Figures and Tables

**Table 1:** Estimated SNR ranges (dB) for the noisy sounds of daily life database, comprising various sounds recorded in the presence of radio noise (two volume levels), speech noise, and rain noise.

	Mic 1	Mic 2	Mic 3	Mic 4
Slamming cupboard door	19–25	23–32	15–18	18–23
Dropping a cup	15–19	12–20	1.0–1.5	3.5–5.0
Dropping a spoon	3.6–4.4	3.3–5.8	0.9–1.6	3.5–4.9
Tap water	1.2–1.7	2.6–5.2	1.4–2.0	2.6–3.2
Chopping vegetables	9–15	8–18	11–14	12–17
Phone ringing	4.5–6.1	5.8–8.7	4.3–5.7	4.3–6.3
Mixmaster	3.6–4.5	6.8–9.7	17–20	9.5–12
Open microwave	2.9–7.0	3.8–9.1	1.4–2.3	2.9–6.8

**Table 2:** Classification accuracy of seven ICA-based methods for source separation for data comprising clean sounds of daily life data in which noise was artificially mixed, to ensure that noise components were perfectly correlated between channels.

	Mic 1, 20 dB,	Mic 1, 25 dB,
	Mic 2, 15 dB	Mic 2, 20 dB
	Accuracy	Accuracy
ICA_SDL_Apriori_	61.33%	62.83%
ICA_BestMic_	61.72%	62.94%
ICA_LeastNoise_	61.61%	63.94%
ICA_SDL_BestSNR_	58.11%	59.79%
ICA_SDL_LeastNoise_	59.56%	60.72%
ICA_SSA_	65.61%	65.94%
ICA_NISA_	**66.06%**	**66.17%**

**Table 3:** Classification accuracies of various combinations of source separation and sound activity detection methods for one of the microphone configurations of the noisy SDL database, across four different noise configurations. Despite this, source separation methods do provide relative reductions in error rate of up to 7%, 48%, and 2% compared with CSLE alone for the radio normal, rain, and radio loud conditions, respectively.

Method	Radio normal	Rain	Radio loud	Talking
	(Mic 1 and Mic 2)	(Mic 1 and Mic 2)	(Mic 1 and Mic 2)	(Mic 1 and Mic 2)
Baseline (channel with best SNR + Baseline SAD)	44.04%	61.39%	28.58%	39.54%
SAD alone: baseline SAD	56.33%	77.78%	32.92%	46.96%
SAD alone: CSLE	71.96%	97.18%	40.25%	**69.45%**
ICA_SDL_Apriori_	44.54%	61.06%	29.71%	48.75%
ICA_SDL_Apriori_ + CSLE	71.63%	98.01%	41.04%	66.95%
ICA_BestMic_	43.83%	61.30%	29.71%	41.54%
ICA_BestMic_ + CSLE	72.13%	97.08%	41.38%	67.12%
ICA_LeastNoise_	43.04%	60.88%	30.46%	44.83%
ICA_LeastNoise_ + CSLE	72.00%	**98.10%**	41.50%	68.25%
ICA_SDL_BestSNR_	44.46%	60.88%	29.50%	41.63%
ICA_SDL_BestSNR_ + CSLE	73.04%	97.50%	41.08%	67.75%
ICA_SDL_LeastNoise_	43.83%	60.56%	30.42%	45.75%
ICA_SDL_LeastNoise_ + CSLE	**73.79%**	96.81%	40.88%	68.75%
ICA_SSA_	44.08%	61.90%	29.83%	40.92%
ICA_SSA_ + CSLE	71.04%	97.04%	**41.58%**	68.21%

**Table 4:** Classification accuracy of promising system configurations, for all three microphone configurations of the noisy SDL database, across four different noise configurations.

Method	Radio normal	Rain	Radio loud	Talking	Average
	(Mic 1 and Mic 2)	(Mic 1 and Mic 2)	(Mic 1 and Mic 2)	(Mic 1 and Mic 2)	
	(Mic 1 and Mic 3)	(Mic 1 and Mic 3)	(Mic 1 and Mic 3)	(Mic 1 and Mic 3)	
	(Mic 1 and Mic 4)	(Mic 1 and Mic 4)	(Mic 1 and Mic 4)	(Mic 1 and Mic 4)	
Baseline (channel with best SNR)	44.04%	61.39%	28.58%	39.54%	43.39%
	51.25%	61.71%	28.75%	44.61%	46.58%
	49.38%	64.91%	33.17%	44.42%	47.97%

SAD alone: CSLE	71.95%	97.18%	40.25%	**69.46%**	69.71%
	**84.08%**	99.77%	55.21%	79.67%	79.68%
	**79.21%**	**98.66%**	**50.63%**	**73.13%**	**75.41%**

ICA_SDL_Apriori_ + CSLE	71.63%	**98.01%**	41.04%	66.96%	69.41%
	83.46%	**100%**	55.88%	**81.67%**	**80.25%**
	77.88%	98.24%	48.71%	44.79%	67.41%

ICA_SDL_BestSNR_ + CSLE	**73.04%**	97.50%	41.08%	67.75%	**69.84%**
	82.79%	99.07%	**57.17%**	76.94%	78.99%
	76.63%	98.24%	49.00%	69.96%	73.46%

ICA_SSA_ + CSLE	71.04%	97.04%	**41.58%**	68.21%	69.47%
	82.50%	99.07%	55.29%	79.00%	78.97%
	75.00%	97.13%	47.29%	67.46%	71.72%
